# Hepatorenal syndrome: a Nationwide Trend Analysis from 2008 to 2018

**DOI:** 10.1080/07853890.2021.1998595

**Published:** 2021-11-16

**Authors:** Jagmeet Singh, Dushyant Singh Dahiya, Asim Kichloo, Gurdeep Singh, Katayoun Khoshbin, Hafeez Shaka

**Affiliations:** a Department of Nephrology, Guthrie Robert Packer Hospital, Sayre, PA, USA; b Department of Internal Medicine, Central Michigan University College of Medicine, Saginaw, MI, USA; c Department of Internal Medicine, Samaritan Medical Center, Watertown, NY, USA; d Department of Medicine and Endocrinology, Lady of Lourdes Memorial Hospital, Binghamton, NY, USA; e Department of Internal Medicine, John H Stroger Hospital of Cook County, Chicago, IL, USA

**Keywords:** Hepatorenal syndrome, liver cirrhosis, nationwide inpatient sample, trends, mortality, outcomes

## Abstract

**Objective:**

This study was designed to assess the epidemiological trends and outcomes associated with Hepatorenal Syndrome (HRS).

**Methods:**

This retrospective interrupted trend study used the Nationwide Inpatient Sample (NIS) database for the years 2008, 2012, 2014, 20z16 and 2018 to identify adult (≥18 years) hospitalizations with a primary diagnosis of HRS. We determined epidemiological characteristics and trends for HRS hospitalizations. Additionally, we also calculated the inpatient mortality, mean length of stay (LOS) and mean total hospital charge (THC) using a multivariate regression trend analysis.

**Results:**

There was an increase in the total number of HRS hospitalizations from 22,864 in 2008 to 42,985 in 2018 with a trend towards increasing hospitalizations (*p*-trend <.001). The mean age for these hospitalizations ranged from 57.4–59.0 years with a significantly rising trend (*p*-trend <.001). Although the majority of HRS hospitalizations were men, we observed a trend towards increasing hospitalizations for women with an increase from 35.7% in 2008 to 39% in 2018 (*p*-trend <.001). Additionally, Whites made up a majority of the sample size (Table 1). After a multivariate regression trend analysis, we found a statistically significant trend towards declining inpatient mortality from 36.2% in 2008 to 25.7% in 2018 (*p*-trend <.001) for HRS hospitalizations (Table 2). We did not find a statistically significant trend for LOS and THC.

**Conclusion:**

Total hospitalizations, hospitalizations for women and the mean age for HRS hospitalizations were on the rise between 2008 and 2018. However, the inpatient mortality declined.

**KEY MESSAGES**
- In the United States, there was a trend towards increasing hospitalizations and mean age for HRS.
- Although a male predominance was noted, HRS hospitalizations for women were on the rise.
- The inpatient mortality for HRS hospitalizations was on a decline and may indicate significant improvements in management.

**Table 1.** Biodemographic characteristics of hospitalizations for Hepatorenal Syndrome (HRS).

	Year					
Variable	2008	2010	2012	2014	2016	2018
Hospitalizations	22,684	27,267	29,765	35,270	39,860	42,985
Mean age (years)	57.4	57.7	58.3	58.9	58.8	59.0
Women (%)	35.7	36.4	35.3	37.1	38.8	39.0
Racial distribution (%)						
White	54.4	62.1	62.8	63.3	64.6	64.8
Black	8.0	10.9	10.2	10.3	10.1	9.0
Hispanic	12.4	12.9	14.0	14.7	14.0	15.7
Others	25.2	14.1	13.0	11.7	11.3	10.5
Insurance type (%)						
Medicaid	36.4	39.7	40.6	43.1	43.4	43.7
Medicare	22.1	21.5	22.3	24.4	25.4	25.2
Private	34.7	31.8	29.8	27.5	26.9	26.1
Uninsured	6.8	7.0	7.3	5.0	4.3	5.0

**Table 2.** Outcomes of hospitalizations for hepatorenal syndrome (HRS).

	Year					
Outcome	2008	2010	2012	2014	2016	2018
Inpatient mortality (%)	36.2	33.8	32.3	29.5	27.8	25.7
Mean length of stay (days)	11.2	11.3	11.1	10.9	10.9	10.8
Mean total hospital cost ($)	36,322	35,486	35,832	34,870	36,922	36,510

## Introduction

Hepatorenal Syndrome (HRS) is a unique form of renal dysfunction in individuals with advanced liver disease or decompensated cirrhosis, and, on occasion, fulminant hepatitis with portal hypertension and ascites [1]. It is characterized by impairment of renal function without significant histological changes [2]. In 2007, HRS was classified into two distinct subtypes which included HRS-1 and HRS-2 [3]. HRS-1 encompassed patients with rapidly worsening of renal function secondary to a precipitating event, whereas HRS-2 was characterized by a slow progression towards renal dysfunction without an obvious trigger [3]. However, this terminology was revised in 2015 by the International Club of Ascites (ICA) to reclassify HRS into HRS-AKI (acute kidney injury) and HRS-NAKI (non-acute kidney injury) based on serum creatinine, glomerular filtration rate (GFR) and urine output [4].

The exact pathophysiological mechanism implicated in the development of HRS is not completely understood and an area of active research, but it is believed to be secondary to renal vasoconstriction and systemic inflammation leading to impairment in renal function [5]. In patients with advanced liver disease, the development of portal hypertension leads to splanchnic vasodilation due to excessive production of vasodilators, particularly nitrous oxide [6]. This causes a significant decrease in the systemic vascular resistance thereby promoting the activation of hypotension-induced vasoconstrictor systems such as the renin-angiotensin-aldosterone system (RAAS) and Endothelin [7]. As a result, renal vasoconstriction ensues leading to decreased renal perfusion and HRS. Additionally, increased cardiac output in patients with progressive liver disease may also result in high-output cardiac failure, which induces renal vasoconstriction [2]. Furthermore, recent literature has suggested that systemic inflammation may also have a key role to play. In cirrhotics, bacterial translocation (usually gram-negative) and endotoxemia can activate the inflammatory cascade due to the release of pro-inflammatory cytokines after recognition of bacterial by-products [pathogen-associated molecular patterns (PAMS)] by immune cells [8]. This causes splanchnic vasodilation and cardiomyocyte dysfunction leading to decreased effective arterial blood volume and activation of homeostatic neurohormonal mechanisms which, in turn, decrease renal perfusion and promote the development of HRS [8].

A meta-analysis by Thompson et al. in 2020 revealed that since 2002 there has been no improvement in survival and reversal rates for HRS [9]. Therefore, it is essential to prevent HRS to decrease mortality and improve outcomes [9]. Although HRS is a relatively well-studied disease entity, the exact incidence and prevalence in the cirrhotic population are currently unknown. However, it is believed that it may be more common than anticipated [10]. Additionally, there is a significant paucity of literature on hospitalizations secondary to HRS. Therefore, this study was designed to help fill these gaps in knowledge. Through this study, we investigated the epidemiological trends of HRS hospitalizations between 2008 and 2018. Furthermore, we assessed inpatient mortality, and the burden of the disease on the United States (US) healthcare system in terms of mean length of stay (LOS) and mean total hospital charge (THC), as well as their trends from 2008 to 2018.

## Materials and methods

### Design and data source

This was a retrospective interrupted trends study which utilized the National Inpatient Sample (NIS) to identify all adult (≥18 years) hospitalizations with a primary discharge diagnosis of HRS between 2008 and 2018. The NIS, part of the Healthcare Cost and Utilization Project (HCUP) databases, is one of the largest and most diverse inpatient databases available in the US, encompassing 97% of the US population [11]. From all inpatient stays, the NIS approximates a 20% stratified sample which is weighted to obtain national estimates [12]. Hence, it is ideal to investigate hospitalization characteristics and adverse outcomes which are applicable to all hospitals across the US. Furthermore, the NIS uses the International Classification of Diseases/Procedure Coding System (ICD/PCS) codes to store data on all inpatient admissions. In our study, the ICD-9 (prior to September 2015) and ICD-10 (after October 2015) codes for HRS were used for analysis. The diagnosis codes within the NIS are divided into two distinct categories, namely the principal diagnosis, and secondary diagnoses. The principal diagnosis for our study was HRS, while the secondary diagnoses were any ICD-9 or ICD-10 codes other than the principal diagnosis.

### Study population

The NIS database was investigated for hospitalizations with a primary principal discharge diagnosis of HRS using ICD codes (572.4 and K76.7) for 2008, 2010, 2012, 2014, 2016 and 2018 study years. Individuals ≤18 years old and those who developed post-procedural HRS were excluded from the study.

### Outcome measures

For the study years, the demographic trends for HRS hospitalizations were highlighted. We also obtained the total hospitalizations for HRS during each calendar year. Using multivariate logistic trend analysis, the inpatient mortality rate, mean LOS and mean THC were calculated. Using 2018 as the reference point, the THC was obtained using the HCUP cost-to-charge ratio files and adjusted for inflation using the Medical Expenditure Panel Survey index for hospital care [13,14].

### Statistical analysis

Stata^®^ Version 16 software (StataCorp, Texas, USA) was used for statistical analysis. All demographic and outcome analyses were performed using weighted samples to obtain national estimates, following HCUP regulations for utilization of the NIS database. A multivariate regression analysis was used to calculate inpatient mortality, mean LOS, and mean THC following adjustment for age, sex, race, Charlson comorbidity index, insurance type, mean household income, and hospital characteristics. All *p*-values were 2 sided, with .05 set as the threshold for statistical significance.

### Ethical considerations

The NIS database lacks patient-specific identifiers. Therefore, our study did not require Institutional Review Board (IRB) approval as per the guideline put forth by our institutional IRB for analysis of HCUP databases.

## Results

### Epidemiological characteristics and trends

**Figure 1.**
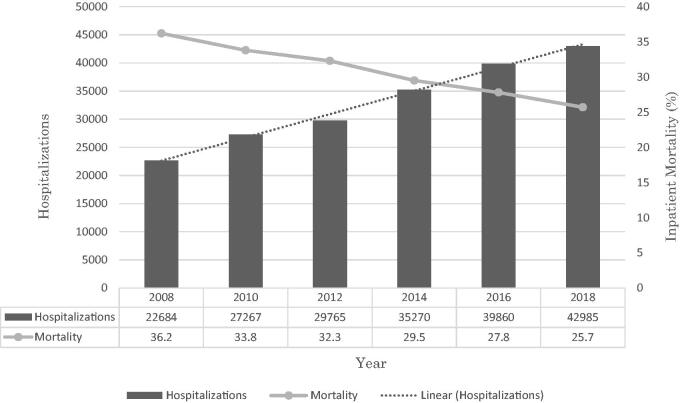
Trends for hepatorenal syndrome (HRS) hospitalizations and inpatient mortality.

We noted an increasing trend for the total number of HRS hospitalizations from 22,864 in 2008 to 42,985 in 2018 (*p*-trend <.001) (Figure 1). The mean age for the study period ranged from 57.4–59.0 years with a statistically significantly rising trend for mean age (*p*-trend <.001) (Table 1). Additionally, a substantial proportion of HRS hospitalizations were for men; however, we noted a trend towards increasing hospitalizations for women from 35.7% in 2008 to 39% in 2018 (*p*-trend <.001). Furthermore, racial differences were also noted for HRS hospitalizations with Whites making up a majority of the cohort for the study period (Table 1).

### Inpatient mortality and trends

After a multivariate regression trend analysis accounting for demographic and hospitalization confounders, there was a statistically significant trend towards declining inpatient mortality from 36.2% in 2008 to 25.7% in 2018 (*p*-trend <.001) for HRS hospitalizations (Table 2).

### Hospitalization characteristics and trends

For the study period, the mean LOS for HRS hospitalizations was noted to be 11.2 d and 10.8 d in 2008 and 2018, respectively. The mean THC for these hospitalizations was $36,322 in 2008 and $36,510 in 2018. However, after a multivariate regression trend analysis, we did not observe a statistically significant trend for THC and LOS between 2008 and 2018. From a payment perspective, Medicaid was the largest insurer (Table 1) for HRS hospitalizations during the study period; however, there was a trend towards increasing the use of Medicare (*p*-trend <.001).

## Discussion

HRS, a well-known complication of advanced liver disease/liver cirrhosis, is characterized by severe renal dysfunction [1,2]. It is associated with high mortality rates and an overall poor prognosis in patients with liver cirrhosis, often requiring liver transplantation (LT). Additionally, the presence of AKI, such as that seen in patients with HRS, in a setting of acute-on-chronic liver failure is associated with significant 90-day mortality [15,16]. In this study, we noted a rise in the total number of HRS hospitalizations between 2008 and 2018; however, inpatient mortality declined from 36.2% in 2008 to 25.7% in 2018. Furthermore, most HRS hospitalizations were for White men. From a healthcare utilization standpoint for HRS hospitalizations, we did not find a statistically significant trend for mean LOS and mean THC. Early identification, diagnosis, and evidenced-based goal-directed management of HRS, particularly in an inpatient setting, is paramount to prevent inpatient mortality, and improve both renal and mortality outcomes [17–19].

### Epidemiology of hepatorenal syndrome hospitalizations

The exact incidence and prevalence of HRS in cirrhotics are currently unknown. Before the introduction of an official diagnostic criterion by ICA, Gines et al. noted the incidence of HRS to be 18% at 1-year and 39% at 5-years for patients with liver cirrhosis and ascites [20]. In 2010, using the revised diagnostic criteria proposed by ICA, a prospective study reported an incidence rate of 7.6% for HRS [21]. Additionally, the prevalence rate of HRS in cirrhotics with ascites is known to range from 13–45.8% [2]. In our study, there was a rising trend for the total number of HRS hospitalizations from 22,864 in 2008 to 42,985 in 2018 (*p*-trend <.001). The exact reason for rising hospitalization rates is currently unknown, but may, in part, be due to changes in the diagnostic criteria and increased awareness of HRS leading to early identification and management [22,23].

HRS is known to affect individuals in their sixth or seventh decade of life with a mean age of 54 years [16]. In our study, the mean was noted to range from 57.4–59.0 years with a rising trend (Table 1). This increasing trend may reflect improved survival secondary to evidenced-based goal-directed protocolized management. Furthermore, we noted a significant male predominance for HRS during the study period however, HRS hospitalizations for females were noted to have an increasing trend from 35.7% in 2008 to 39% in 2018. The exact reason for this finding among females is currently unknown but may be due to a rising prevalence of liver cirrhosis (alcoholic and non-alcoholic) complicated by HRS in females. However, additional large, prospective, multi-center studies are needed to further investigate this finding.

From a racial perspective, significant differences were observed. Whites made up a majority of the study cohort, followed by Hispanics, Blacks and other races (Table 1). These findings were consistent with prior research and may be explained by a similar ethnic distribution of liver cirrhosis which is the underlying disease entity leading to HRS [22,24].

### Inpatient mortality for hepatorenal syndrome hospitalizations

As discussed earlier, HRS is associated with significant morbidity, mortality, and poor prognosis, particularly in patients admitted to the intensive care unit [17–19,25,26]. The model for end-stage liver disease (MELD) scoring system is used to accurately assess prognosis in patients with advanced liver disease [27,28]. Another commonly scoring system, namely Child–Turcotte–Pugh (CTP) score, may also be used to predict mortality and has shown to be superior to the MELD score in cirrhotics with HRS admitted to tertiary teaching hospitals [29]. Patients with type 1 HRS usually have a MELD score ≥20 and a median survival time of 1 month. However, patients with type 2 HRS with a MELD score <20 reportedly have a median survival time of 11 months and those with MELD score ≥20 were noted to have a survival time of 3 months [30]. A retrospective analysis by Jamil et al. noted an inpatient mortality rate of 36.9% for patients with HRS with 8.9% discharged to hospice care [31]. In this study, impatient morality for HRS hospitalizations had a declining trend from 36.2% in 2008 to 25.7% in 2018 (Table 2) after a multivariate regression trend analysis adjusting for demographic characteristics and hospitalization confounders. The exact reason for this decreasing mortality is unknown, but it may be due to early diagnosis and protocolized management of HRS. Additionally, optimizing medical management for liver transplants and the rising rate of liver transplants in the US from 2010 to 2019 may also have a role to play [32–34].

### Burden of hepatorenal syndrome hospitalizations on the United States healthcare system

HRS not only places a significant financial burden on individuals but also on the US healthcare system with an estimated total annual direct medical cost burden of $3–$3.8 billion despite limited treatment options [31,35]. Additionally, patients with HRS eventually require LT which is expected to further increase costs, an overall length of hospital stay, and healthcare resource utilization. Studies estimating the THC for HRS hospitalizations using the NIS database are fairly limited. In our study, the mean THC for HRS hospitalizations was estimated to be $36,322 in 2008 compared to $36,510 in 2018. Furthermore, the mean LOS was noted to be 11.2 d at the start of the study in 2008 and 10.8 d at the end of the study period in 2018. After a multivariate regression trend analysis adjusting for demographic and hospitalization confounders, we did not observe a statistically significant trend for mean LOS and mean THC. The exact reason for the absence of these trends is unknown, but may, in part, be due to a standardized, optimized, and protocol-based treatment approach for HRS hospitalizations.

Moreover, literature reports that patients with commercial insurance are more likely to have higher healthcare costs and readmission rates compared to those with Medicare [35]. In our study, from a pure payment perspective, Medicaid was the largest insurer (Table 1) for HRS hospitalizations between 2008 and 2018. However, after a multivariate regression trend analysis, we observed an increasing trend for utilization of Medicare for HRS hospitalizations. This finding is consistent with the rising trend of mean age noted in our study as patients above the age of 65 would be using Medicaid to cover hospitalization costs.

## Strength and limitations

A key strength of this study is the study population, which is derived from one of the largest, publicly available, multi-ethnic databases available in the US. The NIS database has information on inpatient hospitalizations from across the US. Hence, the results of our study are valid for almost all hospitals in the US. Additionally, the study design, retrospective interrupted, is ideal to assess the trends of the disease entity in question. Moreover, in this study, we assess a wide range of HRS parameters including epidemiological characteristics, adverse outcomes, and the burden of the disease on the US healthcare system, which allows for a more comprehensive analysis, and the addition of meaningful information in the literature.

However, we do acknowledge all the limitations associated with our study. The NIS database does not contain information on the onset of symptoms, disease severity, time to establish the diagnosis, diagnostic tests used, hospital course and the treatment aspects of the disease. The NIS database also does not distinguish between the types of HRS and therefore, we were unable to perform an analysis for the individual types. Furthermore, due to the retrospective study design, it is amenable to all biases associated with retrospective studies. Additionally, a patient discharged with a primary diagnosis of HRS on numerous occasions may have been included multiple times within the dataset as the NIS uses discharge diagnosis, rather than individual patient information, to gather information on the hospitalizations. Finally, the NIS is an administrative database. Hence, the possibility of human errors during the coding process cannot be ruled out. Despite these limitations, we believe that the large sample size, retrospective interrupted-trend study design, and detailed analysis help us better understand HRS. With this study, we aim to encourage intellectual conversation and promote future research on HRS.

## Conclusion

HRS is a well-known complication of advanced liver disease or cirrhosis; however, there is a significant paucity of data on the disease entity, particularly in an inpatient setting. In this study, we noted a rising trend of HRS hospitalizations between 2008 and 2018. However, inpatient mortality declined significantly from 36.2% in 2008 to 25.7% in 2018 after a multivariate regression trend analysis adjusting for demographic and hospital characteristics. The study population was predominantly male with a mean age ranging from 57.4–59.0 years with a trend towards increasing mean age. We also noted a trend towards increasing hospitalizations for females. Furthermore, in 2018, the mean LOS was noted to be 10.8 d, and the mean THC was estimated to be $36,322, but we did not find a statistically significant trend for mean LOS and THC between 2008 and 2018. Medicaid was the largest insurer for the study period; however, there was a trend towards increasing Medicare use. In conclusion, we advocate for the need for additional large, multi-center prospective studies to further investigate the demographic characteristics, associations, and outcomes of HRS.

## Ethical approval

Our institution does not require ethical approval for NIS database studies.

## Authorship statement

Jagmeet Singh and Dushyant Singh Dahiya are credited with substantial contribution to the design of the work, acquisition, and interpretation of the data, drafting the manuscript, revision of important intellectual content, final approval of the version published, and agreement of accountability for all aspects of the work. Asim Kichloo, Gurdeep Singh and Katayoun Khoshbin are credited with substantial contribution to the interpretation of data, literature review of all sections discussed, final approval of the version published, and agreement of accountability for all aspects of the work. Hafeez Shaka is credited with the literature review of all sections discussed, final approval of the version published, and agreement of accountability for all aspects of the work.

## Data Availability

We analyzed the NIS database from 2008 to 2018, available online at http://www.hcup-us.ahrq.gov. The NIS is a large, multi-ethnic, publicly available all-payer inpatient care database in the US containing data on more than seven million hospital stays yearly. Its large sample size is ideal for developing national and regional estimates and enables analyses of rare conditions, uncommon treatments, and special populations.
